# Fabrication, optimization, and characterization of noble silver nanoparticles from sugarcane leaf (*Saccharum officinarum*) extract for antifungal application

**DOI:** 10.1007/s13205-017-0749-y

**Published:** 2017-06-08

**Authors:** Manikandan Velu, Jeong-Ho Lee, Woo-Suk Chang, Nanh Lovanh, Yool-Jin Park, Palaniyappan Jayanthi, Velmurugan Palanivel, Byung-Taek Oh

**Affiliations:** 1 Division of Biotechnology, Advanced Institute of Environment and Bioscience, College of Environmental and Bioresource Sciences, Chonbuk National University, Iksan, Jeonbuk 54596 South Korea; 2 Department of Environmental Science, Periyar University, Salem, Tamil Nadu 636011 India; 3 Sunchang Research Institute of Health and Longevity, Sunchang, Jeonbuk 56015 South Korea; 4 Department of Biology, University of Texas-Arlington, Arlington, TX 76019 USA; 5 USDA-ARS, AWMRU, 230 Bennett Lane, Bowling Green, KY 42104 USA; 6 Department of Ecology Landscape Architecture-Design, College of Environmental and Bioresource Sciences, Chonbuk National University, Iksan, Jeonbuk 54596 South Korea; 7 Plant Medical Research Center, College of Agricultural and Life Sciences, Chonbuk National University, Jeonju, Jeonbuk 54896 South Korea

**Keywords:** Silver nanoparticles, Optimization, Green synthesis, Characterization, Antifungal

## Abstract

Metal nanoparticles obtained from green route are gaining significant prominence as a result of their potential applications in nanomedicine and material engineering. Overall metal nanoparticles studied, silver nanoparticles (AgNPs) clutch prominent place in nanoparticles research field. Herein, we have reported the green synthesis of *Saccharum officinarum* leaf biomass extract-mediated synthesis of AgNPs. Initial nanoparticle production was confirmed by visual observation as color change from light yellow to bright brown color with yellow shade and spectrophotometrically at 450 nm and the various reaction conditions were optimized. The FTIR spectra of the biomass extract and synthesized AgNPs authorized the presence of phyto constituents as capping agent. The High Resolution-Transmission Electron Microscopy (HR-TEM) analyses confirm the morphology and the average particle size of AgNPs as ~28.2 nm. The crystalline nature oxide state and mean particle diameter of AgNPs were confirmed by X-ray diffraction (XRD) analysis, Selected Area Electron Diffraction (SAED) pattern and face-centered cubic (FCC). The obtained AgNPs show moderate to good antifungal activity against *Phytophthora capsici*, *Colletotrichum acutatum* and *Cladosporium fulvum* as 10, 12 and 14 mm zones of inhibition against synthesized AgNPs at 250 μg/well, respectively.

## Introduction

Nanotechnology is an emerging field of science and technology utilized for manufacturing of new materials with different structural and physical properties (Logeswari et al. 2015). The nanomaterials are applied in various aspects such as cosmetics, biomedical, food packaging, drug-gene delivery, environment, optics, chemical industries, electronics, space industries, energy science, photo electrochemistry and catalysis (Ahmed et al. 2016a, b). The application depends on the size (1 to 100 nm) and shape (spherical, circular, hexagonal, etc.,) of the particle (Pattanayak et al. 2015). Particularly, the platinum, gold and silver nanoparticles (AgNPs) are recognized noble metals significantly applied in magnetic, optoelectronics, electronics and information storage (Banerjee et al. 2014). Among the noble metals, AgNPs fetch a prominent place due to its unique properties such as good conductivity, chemical stability, catalytic and most importantly pharmacological aspects such as antibacterial, anti fungal, anti-viral, and anti-inflammatory activities (Velmurugan et al. 2015). However, the same material was utilized in various fields like soaps, detergents, cosmetics, toothpaste, shampoos, and pharmaceutical products too (Banerjee et al. 2014). Earlier reports described that the AgNPs are synthesized through various techniques like electrochemical, aerosol technologies, ultraviolet irradiation, photochemical methods, ultrasonic fields, and laser ablation techniques. However, they used hazardous chemicals and consumed high energy for low conversion materials (Narayanan and Sakthivel 2011; Pattanayak et al. 2015). At this juncture, there is an increase in the interest for synthesis of AgNPs through efficient, alternative, and eco-friendly way by green route. In recent years, AgNPs field of research is focusing on plant and its various part (leafs, fruits, seeds, latex, and peel) extracts particularly the leaf-mediated synthesis was immensely studied *Alternanthera dentate* (Nakkala et al. 2014), *Abutilon indicum* (Sadeghi and Gholamhoseinpoor 2015), *Ziziphora tenuior* (Ulug et al. 2015), *Ficus carica* (Geetha et al. 2014), *Cymbopogan citrates* (Masurkar et al. 2011), *Acalypha indica* (Krishnaraj et al. 2010), *Premna herbacea* (Kumar et al. 2013), *Centella asiatica* (Rout et al. 2013), *Brassica rapa* (Narayanan and Park 2014), *Coccinia indica* (Ashokkumar et al. 2013), *Vitex negundo* (Zargar et al. 2011), *Melia dubia* (Kathiravan et al. 2014), *Portulaca oleracea* (Firdhouse and Lalitha 2012), *Pogostemon benghalensis* (Gogoi 2013), *Swietenia mahogany* (Mondal et al. 2011), *Moringa oleifera* (Prasad and Elumalai 2011), *Garcinia mangostana* (Veerasamy et al. 2011), *Eclipta prostrate* (Rajakumar and Rahuman 2011), *Helianthus tuberosus* (Aravinthan et al. 2015), *Solanum nigrum* and *Solanum indicum* (Sengottaiyan et al. 2016a, b) and *Nelumbo nucifera* (Santhoshkumar et al. 2011). However, there is not much study on the leaf biomass-mediated synthesis of AgNPs.

In the present study, AgNPs was synthesized from sugarcane leaf as a mode to utilize the waste sugarcane leaf for the synthesis of AgNPs which has been quite extensively burned in the agricultural field and small utilize as cattle feed (Miura et al. 2013; Lalitha et al. 2013; Chandel et al. 2012), antioxidant activity (Abbas et al. 2013), anti-mutation and nitric oxide-scavenging activities.

## Materials and methods

### Leaf material and extract preparation

Whole dried sugarcane leaves were collected from the local agricultural land (Salem, Tamil Nadu, India) and washed several times in distilled water to remove dirt. 50 g of sugarcane leaf was cut into small pieces, and boiled in 250 mL of sterile nanopure water in 500 ml Erlenmeyer flask for 30 min at 80 °C to obtain the leaves extract, followed by filtration Whatman No. 42 and stored at 4 °C in refrigerator for future experiments.

### Synthesis of AgNPs

For synthesis of AgNPs using the leaves extract, 40 mL of 1 mM silver nitrate solution and 5 mL of sugarcane leaf extract was added into 100 mL Erlenmeyer flasks and the reaction was carried out under ambient conditions. The reaction mixture was observed for change in color as validation of AgNPs synthesis. The intensity of color was recorded between 200 to 800 nm on UV–visible spectroscopy (UV–1800 UV–vis spectrophotometer, Shimadzu, Kyoto, Japan).

Optimization of AgNPs production under different reaction conditions includes variations in pH (pH 3, 4, 5, 6, 7, 8, 9, and 10), leaf extract reaction volume (0.5, 1, 1.5, 2, 2.5, 3, 3.5, 4, 4.5, and 5 mL), metal ion solution concentration (0.1, 0.2, 0.3, 0.4, 0.5, 0.6, 0.7, 0.8, 0.9, and 1.0 mM), and time interval (0, 15, 30, 45, 60, 75, 90, 105, 120, 135, 150, 165, 180, 195, 210, 240, and 270 min), respectively. While optimizing each parameter, the other parameters remain stable.

### Production and separation of AgNPs

The AgNPs was isolated from the optimized mixture as follows. The obtained reaction mixture was subjected to centrifugation at 12,000 rpm for 10 min; the pellet was purified using nanopure water and washed repeatedly to ensure better separation of free entities from the AgNPs. The obtained AgNPs were lyophilized and used for further characterization and its antifungal potential.

### Characterization of AgNPs

Fourier transform infrared spectroscopy (Perkin–Elmer FTIR spectrophotometer, Norwalk, CT, USA) spectra of the AgNPs were recorded in the diffuse reflectance mode at a resolution of 4 particles cm^−1^ in KBr pellets. XRD analysis of AgNPs was carried out by Rigaku instrument (40 kV, 30 mA) using CuKα radiation with λ of 1.5406 Å and a nickel monochromator filtering wave operating at a tube voltage of 40 kV and a tube current of 30 mA. The scanning was performed in the 2*θ* range of 5°–90° at 0.04°/min with a time constant of 2 s. Further the morphologies, crystalline natures, and size distributions of the AgNPs were analyzed using high resolution-transmission electron microscopy HR-TEM model (JEOL-2010, Japan).

### Antifungal activity

The antifungal activity of Ag_2_O NPs, bulk metal silver, rind extract, AgNPs blend with rind extract, and standard antifungal (nystatin) were determined by a well diffusion method against the plant pathogenic fungi, Viz., *Phytophthora capsici* (KACC 40475), *Phytophthora drechsleri* (KACC 40190), *Didymella bryoniae* (KACC 40900), *Colletotrichum acutatum* (KACC 40042), and *Cladosporium fulvum* (KCCM 11466) obtained from Korean Agricultural Culture Collection (KACC) and maintained in a Potato Dextrose Agar, cultured in potato dextrose agar medium. Agar wells were made using a sterile juice straw, allowing about 5 mm distance to the edge of the plate. Wells were impregnated with AgNPs at concentrations of 20, 40, and 80 μg/well, bulk metal silver (80 μg/well), gum solution (20 μL/well), and AgNPs blend with extract (20 μL/well). All fungal isolates were individually inoculated on sterile PDA medium with a 6 mm cork borer in two edge of the petri plate. The antifungal activity of jack fruit rind extract and AgNPs was determined according to Velmurugan et al. (2015).

## Result and discussion

### Synthesis of AgNPs

Silver nanoparticles formation in the reaction mixture was typically indicated by the color change (Velmurugan et al. 2016). In our study, the AgNPs exhibits dark brown-colored colloidal solution which elucidates the extracellular synthesis of AgNPs on addition of aqueous silver nitrate solution to the leaves extract. The turn in color from watery to brown occurs due to the Surface Plasmon Resonance (SPR) phenomenon of metal nanoparticles in the reaction mixture (Mondal et al. 2011; Nakkala et al. 2014; Velmurugan et al. 2015). Earlier reports suggests that biomolecules such as fatty acid, alcohol, phytosterols, higher terpenoids, flavonoids, –O– and –C-glycosides, and phenolic acids present in the leaf extract (Singh et al. 2015) play a vital role in the conversion of the ionic form of silver to the metallic nanoparticles. In our study, since the sugarcane leaf is enriched with polyphenols (Wang et al. 2011), we can anticipate that, these polyphenols with its hydroxyl moiety will act as reducing agent and promote the reduction of Ag^+^ ions to Ag atoms (Rodriguez-Leon et al. 2013).

**Fig. 1:**
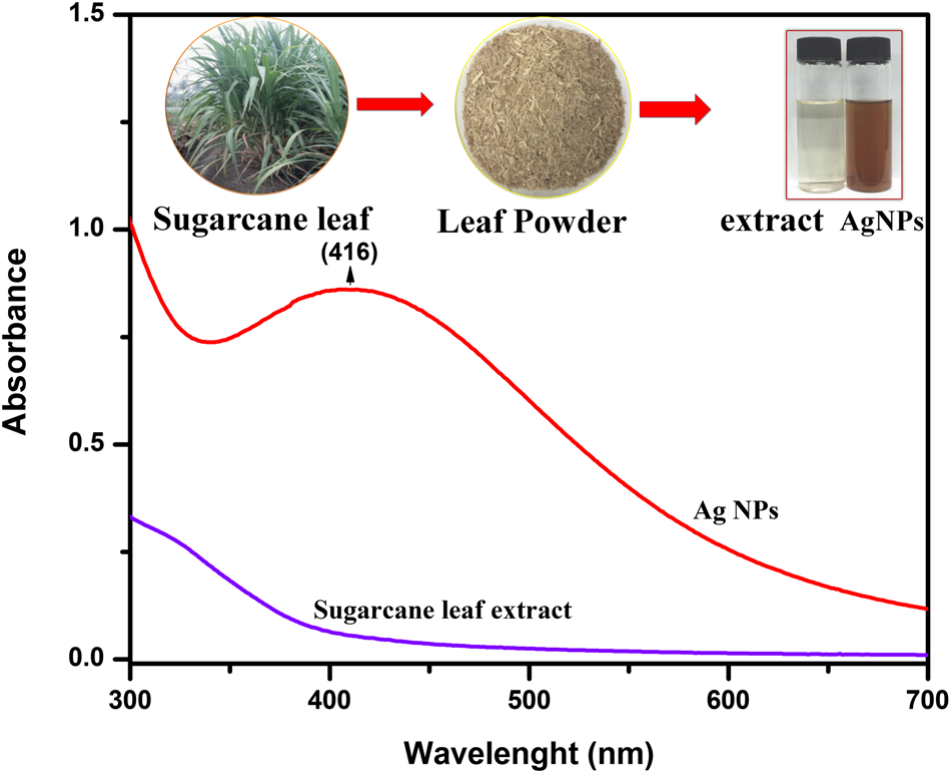
Shows a sugarcane leaf, powder, and color formation in reaction mixture

The formation of AgNPs was observed using UV–visible spectroscopy. Figure 1 represents the UV–visible spectra of AgNPs with its absorption maxima at 416 nm typically in the range of metallic nanoscale silver which is in corroboration with similar findings (Santhoshkumar et al. 2011; Suman et al. 2013; Raja et al. 2015). The shift in absorbance from standard 416 nm may be attributed to the capping of AgNPs by the bioactive constituents in the sugarcane leaf extract (Maria et al. 2015). The single broad SPR peak in the spectra apparently establishes the spherical and polydispersed nature of the AgNPs (Banerjee et al. 2014).

### Factors affecting AgNPs synthesis

**Fig. 2:**
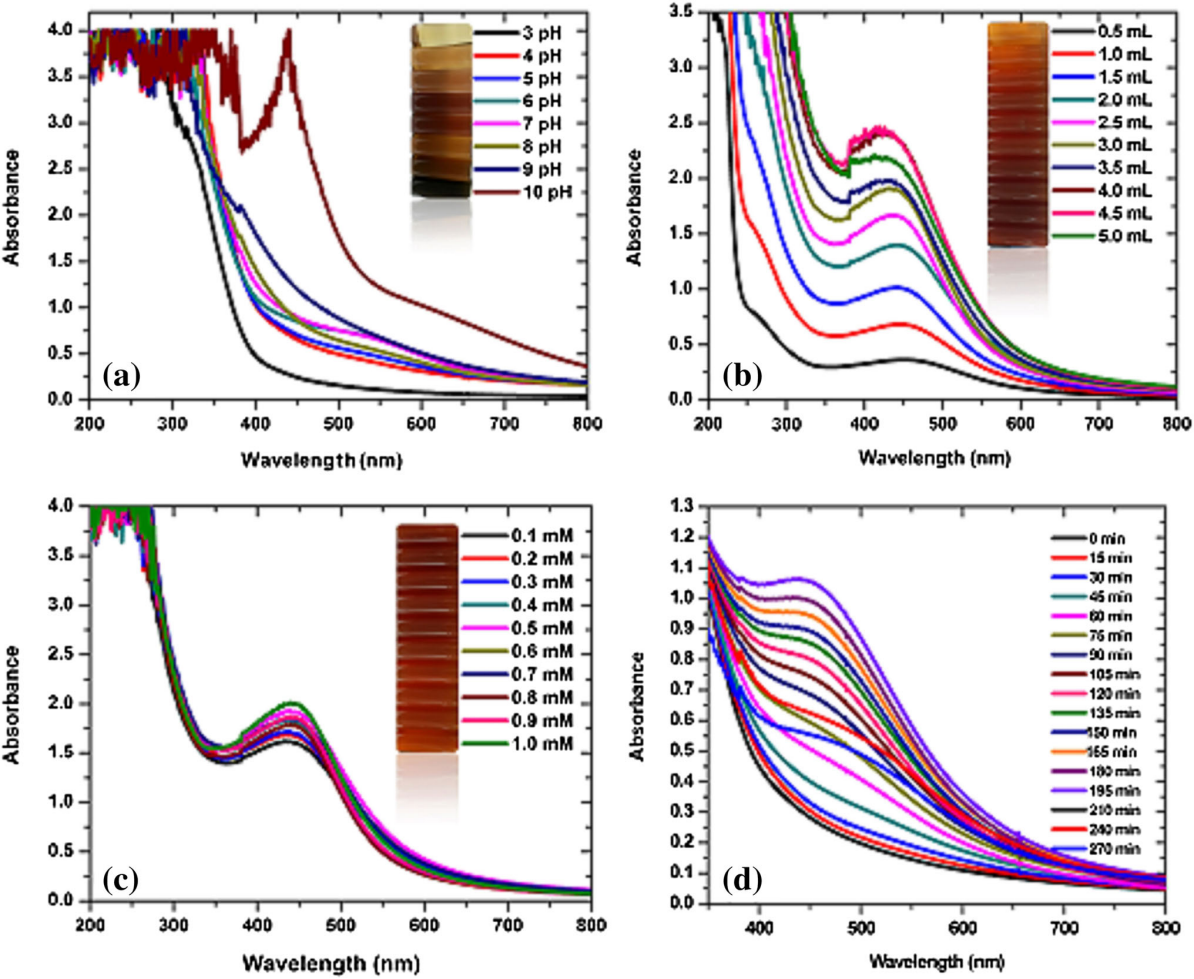
UV–vis absorption spectra of optimizing parameters for the production of AgNPs with different pHs (**a**), ratios of sugarcane leaf extract from 0.5 to 5 mL (**b**), concentration of metal ions Ag^+^ from 0.1 to 1.0 mM (**c**) and different time frame from 0 to 270 min (**d**)

Figure 2a–c shows the absorption spectra of surface plasmon resonance vibrations in the AgNPs synthesised under different reaction parameters such as pH, extract volume, metal ion concentration and the time intervals. Our research provides interesting compilations on assessing under different reaction parameters which had been ascertained as factors affecting the formation of AgNPs (Ahmed et al. 2016a, b).

Earlier reports revealed that the color of reaction mixture and intensity of SPR peaks is pH dependent (Ibrahim 2015). In our study, as shown in Fig. 2a, at acidic pH, there is no SPR peak typically indicating unfavorable condition for AgNPs formation which is in agreement with the earlier findings reported by Veerasamy et al. (2011) and Maria et al. (2015). The typical increase in pH favors the increase in intensity may be due to the large number of functional groups available for binding which facilitates the huge number of AgNPs with small diameters (Veerasamy et al. 2011). The alkaline pH 10.0 only has exhibited SPR peak at nanoscale metallic silver range deciphers that AgNPs synthesis occurs only at strongly alkaline conditions may be due to deposition of hydroxides on AgNPs (Maria et al. 2015).

Figure 2b shows the effect of different volumes of sugarcane leaf extract on the synthesis of AgNPs. The absorption band rises under increasing reaction volume of leaf extract on addition with silver nitrate solution, exhibiting analogous association up to 4.5 mL indicating the higher amount of silver ion reduction whereas on supplementation of 5.0 mL leaf extract contrarily delimits the reduction as indicated by decreased peak absorbance. This contrary relationship exemplifies the constraint exist in reaction due to higher volume of leaf extract to react with precise volume of silver nitrate solution in the reaction mixture (Kalpana et al. 2014).

The maximum absorption was attained at 1.0 mM silver nitrate solution as shown in Fig. 2c indicating much higher oxidation of hydroxyl groups by metal ions. The peak absorbance persistently increased with increase in concentration from 0.1 to 1.0 mM, illustrating the parallel relationship with the metal ion concentration. The increase in intensity with increase in metal ion concentration may be attributed to longitudinal vibrations (Prathna et al. 2011).

Once all the reaction parameters are optimized, synthesis was carried out by the addition of 4 mL sugarcane leaf extract with 40 mL 1.0 mM silver nitrate solution at strongly alkaline pH 10 in 195 min.

### Characterization of silver NPs

#### FTIR spectroscopy

**Fig. 3:**
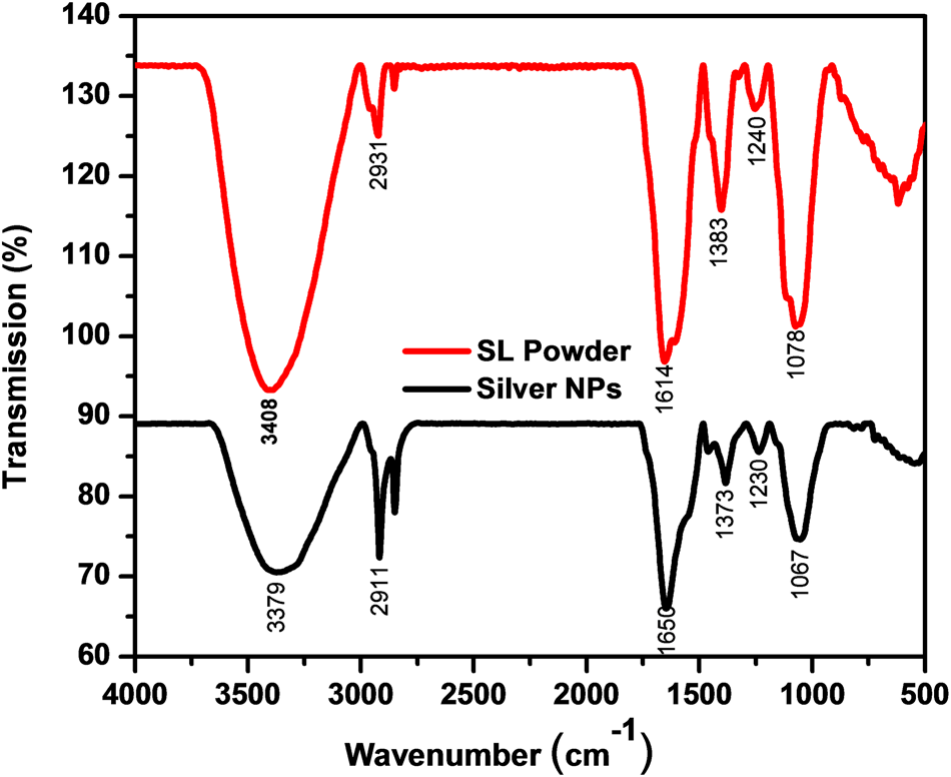
FTIR spectra of the Sugarcane leaf extract powder and AgNPs synthesized at optimum parameters

The fourier transform infrared spectra of the extract and AgNPs were recorded to ascertain the active biomolecules responsible for the reduction of Ag^+^ ions (Fig. 3). The shift in absorbance from 3379, 2911, 1650, 1373, 1230 cm^−1^, and 1373, 1230, 1067 cm^−1^ and 3408, 2931, 1614, 1383, 1240 and 1078 cm^−1^, respectively, in the absorption spectra of AgNPs indicates the involvement of above various active biomolecules like proteins and phenols in the reduction of Ag^+^ ions to Ag atoms. The absorption peak at 3408 cm^−1^ is due to the N–H stretch vibrations of primary amines and the strong peak at 2931 cm^−1^ corresponding to stretch of O–H of carboxylic acids and its derivatives. The strong peak at 1614 cm^−1^ occurs due to the –NH_2_ bending vibrations of aliphatic amines. The band existence at 1383 cm^−1^ is due to the in-plane bending vibrations of –O–H group. The peak 1240 cm^−1^ corresponds to C–N stretching vibration of aliphatic amine (Balashanmugam and Kalaichelvan 2015). The strong peak at 1078 cm^−1^ is attributed to C–O stretching possibly due to the presence of carboxylic acid group. Thus, the presence of –NH, –OH, and C–O confirms the presence of proteins and carboxylic acid derivatives, phenols in the extract.

#### HR-TEM analysis

**Fig. 4:**
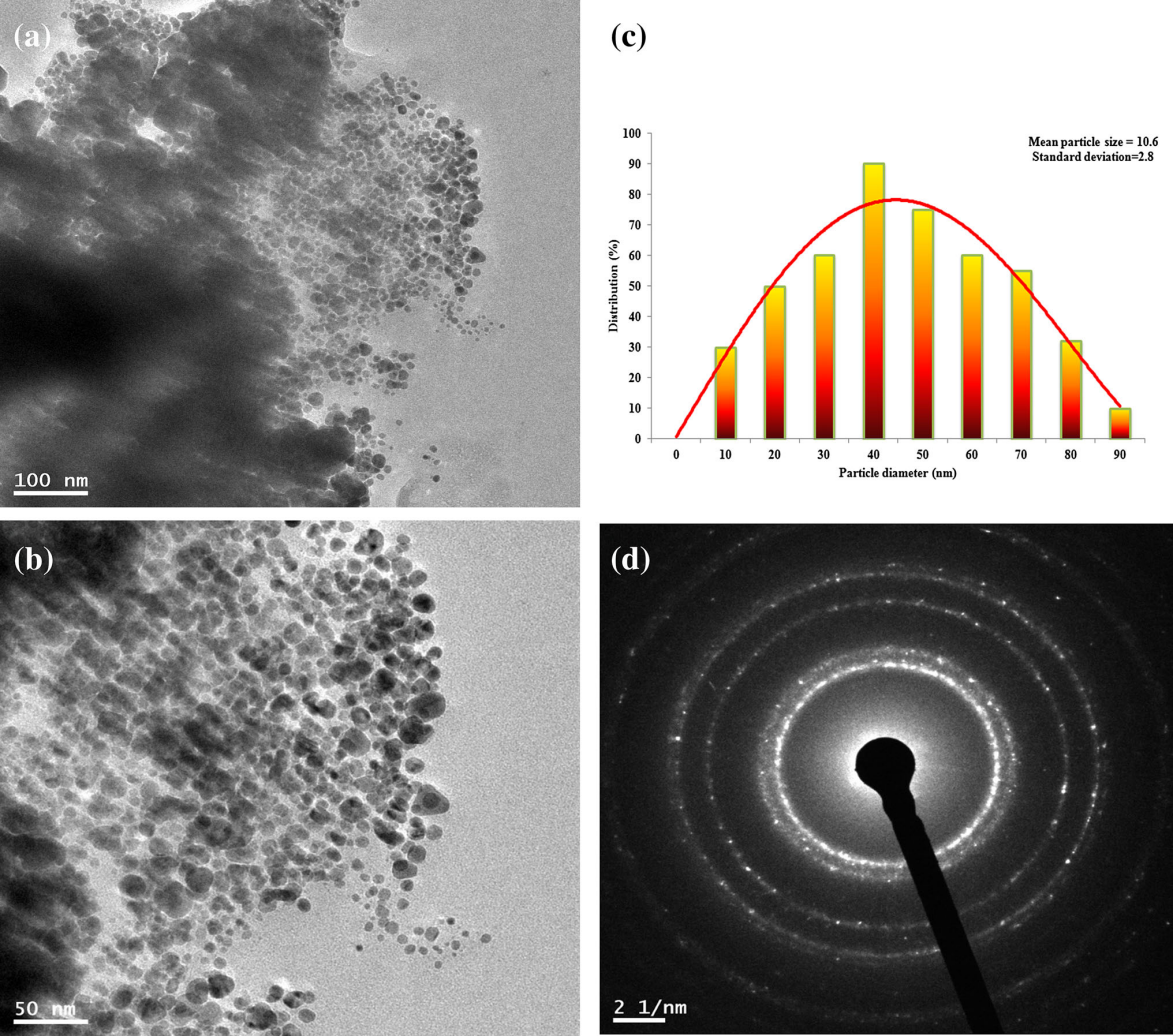
TEM images of AgNPs (**a**) 24.2 nm (100 nm), (**b**) Image of 50 nm, (**c**) particle distribution histogram, (**d**) corresponding SAED pattern for AgNPs

HR-TEM is employed to determine the morphology, size, and shape of the nanoparticles. The shape of the nanoparticles was in spherical and non-uniform with an average size of ~20–50 nm with little aggregation (Fig. 4a, b). TEM micrographs provided further insight into the morphology and particle size distribution profile (Fig. 4c) of the sugarcane leaf extract AgNPs. It has been observed that the AgNPs of smallest particle sizes with the average size of 10.5 ± 8.2 nm. To confirm the crystallinity of Silver NPs, the selected area electron diffraction (SAED) pattern-recorded single particle in the aggregates of all the nanoparticle samples corresponding to a characteristic polycrystalline ring pattern for a face-centered-cubic structure (Fig. 4d) revealed that the synthesized AgNPs were crystalline in nature.

#### XRD

**Fig. 5:**
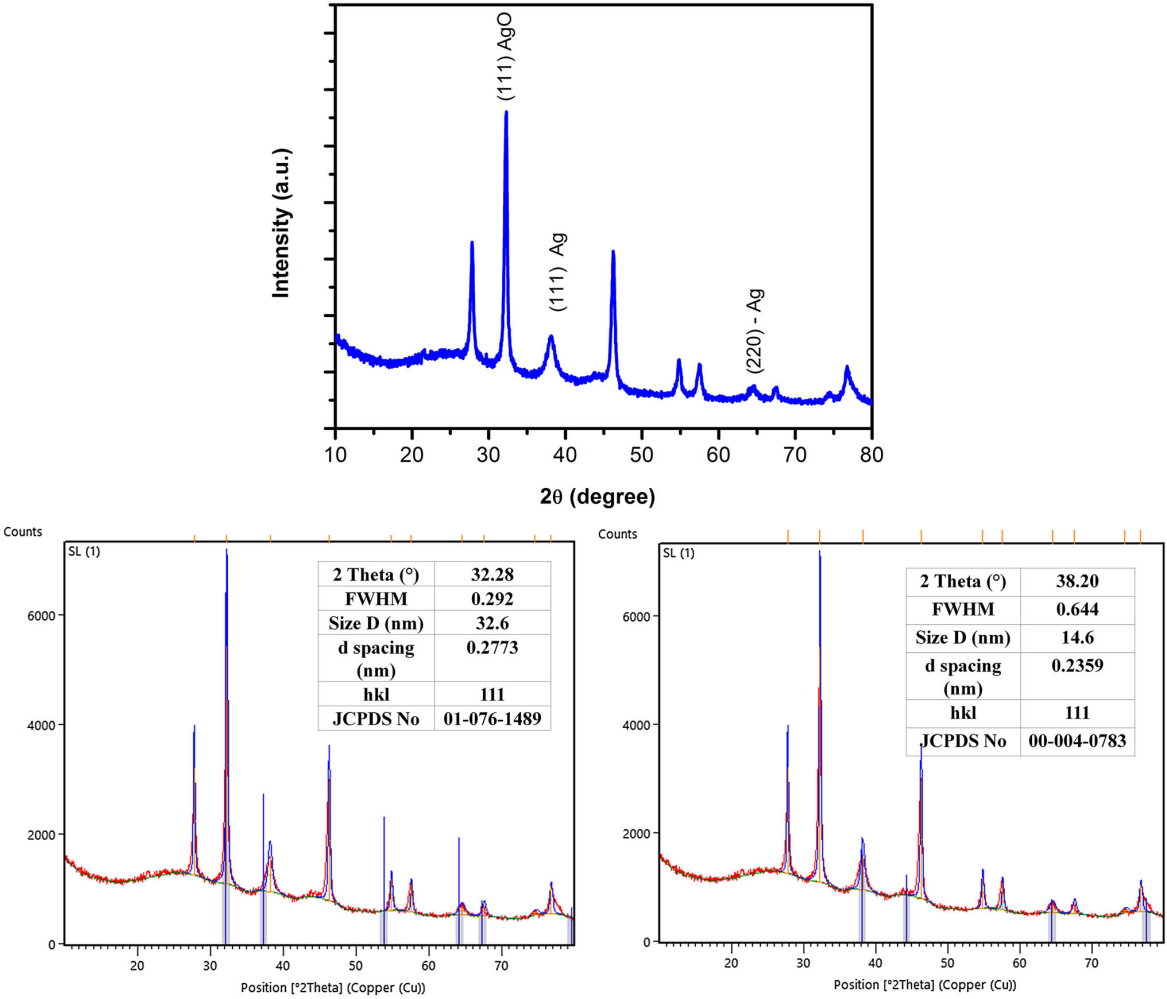
XRD patterns of the AgNPs synthesized at optimum parameters by Sugarcane leaf extract

The X-ray diffraction pattern depicts the crystallinity of AgNPs and the oxide pattern as shown in (Fig. 5). It is apparent that characteristic diffraction peaks at (111), (111), (220), in 2*θ* corresponds to lattices of (32.20°), (38.20°), and (65.00°) planes of pure AgNPs match with that of the standard spectra of silver (JCPDS No.# 00-004-0783), and silver oxide (JCPDS No.# 01-076-1489) (Velmurugan et al. 2016). The result confirms the face-centered-cubic lattice of silver suggesting the biphasic nature of AgNPs (Mallikarjuna et al. 2014; Velmurugan et al. 2016).

XRD analysis, therefore, confirms the face-centered cubic (FCC) configuration of biosynthesised AgNPs using software PANalytical X’Pert HighScore Plus, Version 3.0.3 (Fig. 5). The mean particle diameter of AgNPs was calculated from the XRD pattern using the Scherrer equation:

$$ D = K\lambda /\beta_{1/2} {\text{COS}}\,\theta , $$

where *K* is the shape constant, *λ* is the wavelength of the X-ray, *β*_1/2_ and *u* are the half width of the peak and half of the Bragg’s angle, respectively. The calculated average crystallite size of the AgNPs was found to be 24.2 nm from the breadth of the (111) reflection.

#### Antifungal activity

The in vitro antifungal activity of synthesized AgNPs with commercially available antifungal agent was carried out for the first time against five different plant pathogenic fungi. AgNPs and a combination of extract and AgNPs showed remarkable activity against four plant pathogenic fungi, *C. acutatum*, *P. capsici*, *P. drechsleri,* and *C. fulvum* (Table 1). *P. drechsleri* showed resistance to the commercial antifungal agent nystatin (20 µg/well); however, *D. bryoniae* were resistant to synthesized AgNPs (Table 1).

**Table 1:** Zone of inhibition (mm) of synthesized AgNPs and blend of extract with AgNPs against plant fungal pathogens

S. no.	Strains	Zone of inhibition (ZOI) in mm							
		Extract	AgNPs + extract			Synthesized Ag_2_O NPs			Control
			25 μL	50 μL	100 μL	100 μg	150 μg	200 μg	
1.	*C. acutatum*	–	1	2	4	1	2	4	22
2.	*P. capsici*	–	1	3	5	1	3	5	24
3.	*P. drechsleri*	–	1	3	4	1	3	4	–
4.	*C. fulvum*	–	1	4	4	1	4	4	22
5.	*D. bryoniae*	–	–	–	–	–	–	–	24

## Conclusion

In the present study, we demonstrate a novel eco-friendly, rapid and low cost method for the synthesis of AgNPs in a green chemistry approach by sugarcane leaf extract to make use of an agricultural waste. By avoiding the use of any toxic chemical, as an alternate the bioactive molecules from the extract act as both reducing and capping agents. The synthesized AgNPs were characterized by UV–vis spectroscopy, FTIR, HR-TEM, and XRD techniques. The obtained nanoparticles presented almost spherical shape in the sizes ranging ~10 and 60 nm, with little aggregation. The antifungal activity of AgNPs was investigated against plant pathogenic fungus and it shows moderate activity to few pathogens.

Based on these findings, the current method can be suitable for the industrial scale production of AgNPs from a commonly burning agricultural waste sugarcane leaf.

## References

[CR1] Abbas SR, Ahmad SD, Sabir SM, Shah AH, Awan S, Gohar M, Khan MF, Rao AZ (2013). Antioxidant activity, repair and tolerance of oxidative DNA damage in different cultivars of Sugarcane (*Saccharum officinarum*) leaves. Aust J Crop Sci.

[CR2] Ahmed S, Ahmad M, Swami BL, Ikram S (2016). A review on plants extract mediated synthesis of silver nanoparticles for antimicrobial applications: a green expertise. J Adv Res.

[CR3] Ahmed S, Saifullah Ahmad M, Swami BL, Ikram S (2016). Green synthesis of silver nanoparticles using *Azadirachta indica* aqueous leaf extract. J Radiat Res Appl Sci.

[CR4] Aravinthan A, Govarthanan M, Selvam K, Praburaman L, Selvankuma T, Balamurugan R, Kamala-Kannan S, Kim JH (2015). Sunroot mediated synthesis and characterization of silver nanoparticles and evaluation of its antibacterial and rat splenocyte cytotoxic effects. Int J Nanomed.

[CR5] Ashokkumar S, Ravi S, Kathiravan V (2013). Green synthesis of silver nanoparticles and their structural and optical properties. Int J Curr Res.

[CR6] Balashanmugam P, Kalaichelvan PT (2015). Biosynthesis characterization of silver nanoparticles using *Cassia roxburghii* DC. aqueous extract, and coated on cotton cloth for effective antibacterial activity. Int J Nanomed.

[CR7] Banerjee P, Satapathy M, Mukhopahayay A, Das P (2014). Leaf extract mediated green synthesis of silver nanoparticles from widely available Indian plants: synthesis, characterization, antimicrobial property and toxicity analysis. Bioresour Bioprocess.

[CR8] Chandel AK, da Silva SS, Carvalho W, Singh OV (2012). Sugarcane bagasse and leaves: foreseeable biomass of biofuel and bio-products. J Chem Technol Biotechnol.

[CR9] Firdhouse MJ, Lalitha P (2012). Green synthesis of silver nanoparticles using the aqueous extract of *Portulaca oleracea* (L.). Asian J Pharm Clin Res.

[CR10] Geetha N, Geetha TS, Manonmani P, Thiyagarajan M (2014). Green synthesis of silver nanoparticles using *Cymbopogan citratus* (Dc) Stapf. extract and its antibacterial activity. Aust J Basic Appl Sci.

[CR11] Gogoi SJ (2013). Green synthesis of silver nanoparticles from leaves extract of ethnomedicinal plants-*Pogostemon benghalensis (B) O. Ktz*. Adv Sci Res.

[CR12] Ibrahim HMM (2015). Green synthesis and characterization of silver nanoparticles using banana peel extract and their antimicrobial activity against representative microorganisms. J Radiat Res Appl Sci.

[CR13] Kalpana D, Han JH, Park WS, Lee SM, Wahab R, Lee YS (2014). Green biosynthesis of silver nanoparticles using *Torreya nucifera* and their antibacterial activity. Arab J Chem.

[CR14] Kathiravan V, Ravi S, Ashokkumar S (2014). Synthesis of silver nanoparticles from *Melia dubia* leaf extract and their in vitro anticancer activity. Spectrochim Acta Part A Mol Biomol Spectrosc.

[CR15] Krishnaraj C, Jagan EG, Rajasekar S, Selvakumar P, Kalaichelvan PT, Mohan N (2010). Synthesis of silver nanoparticles using *Acalypha indica* leaf extracts and its antibacterial activity against water borne pathogens. Colloids Surf B Biointerfaces.

[CR16] Kumar S, Daimary RM, Swargiary M, Brahma A, Kumar S, Singh M (2013). Biosynthesis of silver nanoparticles using *Premna herbacea* leaf extract and evaluation of its antimicrobial activity against bacteria causing dysentery. Int J Pharm Bio Sci.

[CR17] Lalitha A, Subbaiya R, Ponmurugan P (2013). Green synthesis of silver nanoparticles from leaf extract *Azhadirachta indica* and to study its anti-bacterial and antioxidant property. Int J Curr Microbiol App Sci.

[CR18] Logeswari P, Silambarasan S, Abraham J (2015). Synthesis of silver nanoparticles using plants extract and analysis of their antimicrobial property. J Saudi Chem Soc.

[CR19] Mallikarjuna K, Sushma NJ, Narasimha G, Manoj L, Raju BDP (2014). Phytochemical fabrication and characterization of silver nanoparticles by using *Pepper* leaf broth. Arabian J Chem.

[CR20] Maria BS, Devadiga A, Kodialbail VS, Saidutta MB (2015). Synthesis of silver nanoparticles using medicinal *Zizyphus xylopyrus* bark extract. Appl Nanosci.

[CR21] Masurkar SA, Chaudhari PR, Shidore VB, Kamble SP (2011). Rapid biosynthesis of silver nanoparticles using *Cymbopogan citratus* (Lemongrass) and its antimicrobial activity. Nano-Micro Lett.

[CR22] Miura T, Niswati A, Swibawa IG, Haryani S, Gunito H, Kaneko N (2013). No tillage and bagasse mulching alter fungal biomass and community structure during decomposition of sugarcane leaf litter in Lampung Province, Sumatra, Indonesia. Soil Biol Biochem.

[CR23] Mondal S, Roy N, Laskar RA, Sk I, Basu S, Mandal D, Begum NA (2011). Biogenic synthesis of Ag, Au and bimetallic Au/Ag alloy nanoparticles using aqueous extract of mahogany (*Swietenia mahogani* JACQ.) leaves. Colloids Surf B Biointerfaces.

[CR24] Nakkala JR, Mata R, Gupta AK, Sadras SR (2014). Biological activities of green silver nanoparticles synthesized with *Acorus calamus* rhizome extract. Eur J Med Chem.

[CR25] Narayanan KB, Park HH (2014). Antifungal activity of silver nanoparticles synthesized using turnip leaf extract (*Brassica rapa* L.) against wood rotting pathogens. Eur J Plant Pathol.

[CR26] Narayanan KB, Sakthivel N (2011). Extracellular synthesis of silver nanoparticles using the leaf extract of *Coleus amboinicus* Lour. Mater Res Bull.

[CR27] Pattanayak S, Mollick MMR, Maity D, Chakraborty S, Dash SK, Chattopadhyay S, Roy S, Chattopadhyay D, Chakraborty M (2015). *Butea monosperma* bark extract mediated green synthesis of silver nanoparticles: characterization and biomedical applications. J Saudi Chem Soc.

[CR28] Prasad TNVKV, Elumalai EK (2011). Biofabrication of Ag nanoparticles using *Moringa oleifera* leaf extract and their antimicrobial activity. Asian Pac J Trop Biomed.

[CR29] Prathna TC, Chandrasekaran N, Raichur AM, Mukherjee A (2011). Biomimetic synthesis of silver nanoparticles by *Citrus limon* (lemon) aqueous extract and theoretical prediction of particle size. Colloids Surf B Biointerfaces.

[CR30] Raja S, Ramesh V, Thivaharan V (2015). Green biosynthesis of silver nanoparticles using *Calliandra haematocephala* leaf extract, their antibacterial activity and hydrogen peroxide sensing capability. Arab J Chem.

[CR31] Rajakumar G, Rahuman AA (2011). Larvicidal activity of synthesized silver nanoparticles using *Eclipta prostrata* leaf extract against filariasis and malaria vectors. Acta Trop.

[CR32] Rodriguez-Leon E, Iñiguez-Palomares R, Navarro RE, Herrera-Urbina R, Tánori J, Iñiguez-Palomares C, Maldonado A (2013). Synthesis of silver nanoparticles using reducing agents obtained from natural sources (Rumex hymenosepalus extracts). Nanoscale Res Lett.

[CR33] Rout ANANDINI, Jena PK, Parida UK, Bindhani BK (2013). Green synthesis of silver nanoparticles using leaves extract of *Centella asiatica* L. For studies against human pathogens. Int J Pharm Bio Sci.

[CR34] Sadeghi B, Gholamhoseinpoor F (2015). A study on the stability and green synthesis of silver nanoparticles using *Ziziphora tenuior* (Zt) extract at room temperature. Spectrochim Acta Part A Mol Biomol Spectrosc.

[CR35] Santhoshkumar T, Rahuman AA, Rajakumar G, Marimuthu S, Bagavan A, Jayaseelan C, Zahir AA, Elango G, Kamaraj C (2011). Synthesis of silver nanoparticles using *Nelumbo nucifera* leaf extract and its larvicidal activity against malaria and filariasis vectors. Parasitol Res.

[CR36] Sengottaiyan A, Aravinthan A, Sudhakar C, Selvam K, Srinivasan P, Govarthanan M, Manoharan K, Selvankumar T (2016). Synthesis and characterization of Solanum nigrum-mediated silver nanoparticles and its protective effect on alloxan-induced diabetic rats. J Nanostruct Chem.

[CR37] Sengottaiyan A, Mythili R, Selvankumar T, Aravinthan A, Kamala-Kannan S, Manoharan K, Thiyagarajan P, Govarthanan M, Kim JH (2016). Green synthesis of silver nanoparticles using *Solanum indicum* L. and their antibacterial, splenocyte cytotoxic potentials. Res Chem Intermed.

[CR38] Singh A, Lal UR, Mukhtar HM, Singh PS, Shah G, Dhawan RK (2015). Phytochemical profile of sugarcane and its potential health aspects. Pharmacogn Rev.

[CR39] Suman TY, Rajasree SR, Kanchana A, Elizabeth SB (2013). Biosynthesis, characterization and cytotoxic effect of plant mediated silver nanoparticles using *Morinda citrifolia* root extract. Colloids Surf B Biointerfaces.

[CR40] Ulug B, Turkdemir MH, Cicek A, Mete A (2015). Role of irradiation in the green synthesis of silver nanoparticles mediated by fig (*Ficus carica*) leaf extract. Spectrochim Acta Part A Mol Biomol Spectrosc.

[CR41] Veerasamy R, Xin TZ, Gunasagaran S, Xiang TFW, Yang EFC, Jeyakumar N, Dhanaraj SA (2011). Biosynthesis of silver nanoparticles using mangosteen leaf extract and evaluation of their antimicrobial activities. J Saudi Chem Soc.

[CR42] Velmurugan P, Cho M, Lim SS, Seo SK, Myung H, Bang KS, Sivakumar S, Cho KM, Oh BT (2015). Phytosynthesis of silver nanoparticles by *Prunus yedoensis* leaf extract and their antimicrobial activity. Mater Lett.

[CR43] Velmurugan P, Shim J, Kim K, Oh BT (2016). *Prunus* × *yedoensis* tree gum mediated synthesis of platinum nanoparticles with antifungal activity against phytopathogens. Mater Lett.

[CR44] Wang BS, Duh PD, Wu SC, Huang MH (2011). Effects of the aqueous extract of sugarcane leaves on antimutation and nitric oxide generation. Food Chem.

[CR45] Zargar M, Hamid AA, Bakar FA, Shamsudin MN, Shameli K, Jahanshiri F, Farahani F (2011). Green synthesis and antibacterial effect of silver nanoparticles using *Vitex negundo* L. Molecules.

